# Dismal management of hypertension at primary level: does it reflect a failure of patients, a failure of the system or a failure of doctors?

**Published:** 2011-08

**Authors:** Ntobeko BA Ntusi

**Affiliations:** Department of Medicine, University of Cape Town and Groote Schuur Hospital, Cape Town, South Africa; Department of Cardiovascular Medicine, University of Oxford and John Radcliffe Hospital, Oxford, United Kingdom

‘My great concern is not whether you have failed, but whether you are content with your failure.’ *Abraham Lincoln*

Hypertension is a major but modifiable risk factor for cardiovascular disease (CVD). About 25% of adults in the world have hypertension and this is expected to increase in the coming years.^1^ In sub-Saharan Africa (SSA), the number of hypertensive adults is projected to rise from 80 million in 2000 to 150 million by 2025.^2^

In SSA, not only is hypertension common, but it is frequently misdiagnosed. This is of great economic relevance as it commonly affects the young, and causes serious complications. 3,4 Persistently elevated blood pressure is associated with poor outcomes, including left ventricular hypertrophy, heart failure, stroke, premature coronary artery disease, chronic kidney disease, intra-cerebral haemorrhage, retinopathy, vascular dementia and acute life-threatening emergencies. Moreover, the management of hypertension remains sub-optimal worldwide.

Data from the National Health and Nutrition Examination Survey (NHANES) and the United States Census Bureau from 1999 to 2000 revealed that only 29 to 31% of adult hypertensives were controlled, with the prevalence of poor control being higher in older people and blacks.^5^ Multiple patient-, system- and physician-related factors contribute to the poor management of hypertension, despite the publication of best-practice guidelines for management of high blood pressure by different professional societies.

In this issue of the journal, Parker and colleagues report on a study conducted in Cape Town, South Africa, showing that these doctors’ knowledge on the management of hypertension and of the South African hypertension guidelines was poor, with 62.5% of the doctors surveyed attempting to treat hypertension to target, and only 50% recommending lifestyle modifications to their patients.^6^ Furthermore, physician inertia was rife, as the participating doctors estimated that about 35% of their hypertensive patients were controlled on the treatment prescribed, and yet therapy was not routinely up-titrated for the majority of patients.

Factors identified by these doctors as impacting on optimal management of hypertension at primary level included poor patient adherence to prescribed treatment, communication difficulties (as the doctors did not always speak the language of the patients), heavy patient workloads and staff shortages, and patient loss to follow up.

## Hypertension in South Africa

Hypertension in South Africa was responsible for 46 888 deaths (9% of all deaths) and 2.4% of disability-adjusted life years in 2000.^7^ Risk factors for high blood pressure in South Africa include low educational attainment, older age, increased weight, excess use of alcohol and a family history of hypertension or stroke.^8^

Hypertension in black South Africans differs in its clinical presentation, epidemiology and complications from that of white South Africans and hypertensive patients from developed countries. Black South Africans, particularly the young, typically have malignant hypertension complicated by hypertensive nephrosclerosis, stroke, hypertensive heart disease, heart failure more commonly, and coronary artery disease less frequently.^8^,^9^ Furthermore, the mortality from stroke is twice as high among blacks compared to other race groups.^10^

## Poor control of hypertension at primary level in South Africa

Poor management of hypertension by despondent primary healthcare workers in South Africa has previously been documented.^11^,^12^ Rayner *et al*.^13^ and Steyn *et al*.^14^ have reported that only 39.8 and 42.1%, respectively, of South African primary healthcare hypertensive patients were adequately controlled. Furthermore, Schoeman and Rayner, from a cross-sectional analysis of South African hypertensive patients in general practice have demonstrated that physician inertia is common, with 30.7% of hypertensive patients on monotherapy, 42.8% on two drugs and only 26.5% receiving more than two agents.^15^

## Risk factors for hypertension

Essential (primary) hypertension is responsible for the majority of cases of elevated blood pressure, where it is found to be more severe and more common in blacks, older people and obese women. High salt intake, excessive alcohol use, physical inactivity and dyslipidaemia remain important additional risk factors. Several other factors are risk factors/causes of secondary hypertension (as summarised in Table 1).

**Table 1.** Risk Factors For Hypertension

*Essential hypertension*	*Secondary hypertension*
More common and more severe in blacks, older people and women	Primary renal disease
High salt intake	Renovascular disease
Hypertension in parents	Primary aldosteronism
Excess alcohol intake	Phaeochromocytoma
Obesity	Cushing’s syndrome
Physical inactivity	Pregnancy
Dyslipidaemia (independent of obesity)	Sleep apnoea
Certain personality traits (e.g. Type A personality)	Drugs (e.g. oral contraceptive pill, non-steroidal anti-inflammatory drugs)
High intake of fructose from sweetened beverages	Other endocrinopathies (e.g. hyperthyroidism, hypothyroidism, hyperparathyroidism, acromegaly, etc)
Multiple genetic polymorphisms	Aortic coarctation

## Refractory hypertension

Multiple factors contribute to poor control of hypertension. Refractory hypertension is an indication for referral of hypertensive patients from primary-care centres to a higher level of care for further investigation and modification of therapeutic strategy. Refractory hypertension is defined as poorly controlled blood pressure in a patient receiving three or more antihypertensive agents prescribed at optimal doses, one of which must be a diuretic.^16^ However, before diagnosing refractory hypertension, it is important to exclude pseudo-resistance, which may emanate from patient non-adherence to prescribed therapy, ‘white coat’ hypertension or inaccurate measurement of blood pressure, among other factors.

Risk factors for refractory hypertension include presence of left ventricular hypertrophy, older age, obesity, African race, chronic kidney disease, diabetes mellitus and obstructive sleep apnoea.^16^ Table 2 summarises the important conditions that need to be considered in the investigation of a patient with refractory hypertension.

**Table 2.** Conditions Associated With Refractory Hypertension

1.	Sub-optimal antihypertensive therapy
2.	Extracellular volume expansion
3.	Poor adherence to medical and dietary therapy
4.	Secondary hypertension
5.	Undiagnosed kidney disease
6.	Primary aldosteronism
7.	Ingestion of substances that elevate blood pressure (alcohol, herbal remedies, oral contraceptive pill, anti-depressant medications, non-steroidal anti-inflammatories)
8.	Office or ‘white coat’ hypertension
9.	Lifestyle and diet (e.g. high salt intake)
10.	Pseudohypertension

## Compelling indications for treatment of hypertension

In the report by Parker and colleagues,^6^ knowledge of the compelling indications for the management of hypertension was found to be quite poor among the primary healthcare doctors studied. The South African hypertension guidelines provide a rational and evidence-based approach for the management of individuals with hypertension.^17^ In the presence of certain conditions, the first-line recommended agents vary, as discussed below.

• Angiotensin converting enzyme (ACE) inhibitors should be first-line agents in the presence of heart failure, asymptomatic left ventricular dysfunction, diabetes mellitus, coronary artery disease and proteinuric kidney disease.

• Angiotensin II receptor blockers (ARBs) have similar indications as ACE inhibitors, and may be preferable where there is electrocardiographic evidence of left ventricular hypertrophy or intolerance to ACE inhibitors.

• Diuretics are the usual recommended first-line therapy in the absence of compelling indications, and are useful agents in the presence of volume overload. Chlorthalidone is the preferred thiazide diuretic for essential hypertension, but is not available in most African countries.

• Calcium channel blockers (CCBs) have no absolute indications, but may be useful for rate control in atrial fibrillation and in patients with angina pectoris. CCBs are preferable to beta-blockers in those with obstructive airway disease.

• Beta-blockers may be considered as first-line treatment (but are usually given in combination with an ACE inhibitor) after acute myocardial infarction, in stable patients with heart failure, asymptomatic left ventricular dysfunction, for rate control in atrial fibrillation, and for symptom control in ischaemic heart disease. In the absence of these conditions, beta-blockers should not be first line or given as monotherapy, especially in patients older than 60 years, where they have been associated with higher rates of stroke, coronary artery disease and all cardiovascular events, compared to other antihypertensives.^18^

• Alpha-blockers are not recommended as first-line agents or as monotherapy because of the increased risk of heart failure and increased cardiovascular events associated with their use.

## Recommendations

Doctors should aim to treat all hypertensive patients to target, as hypertension is a modifiable risk factor for CVD. It is the amount of blood pressure reduction that determines the absolute reduction in cardiovascular risk and not the choice of antihypertensive therapy. Hence, the aim should be to lower blood pressure at all costs. The management strategy should take into account the patient’s individual needs and preferences.

People with hypertension should have the opportunity to make informed decisions about their care and treatment, as this will likely increase their adherence to the prescribed medication. Good communication between healthcare professionals, especially doctors, and patients is essential. Care of people with hypertension needs to be supported by provision of evidence-based information such as the South African hypertension guidelines.^17^

Lifestyle interventions need to be offered to all patients. These include weight loss, smoking cessation, moderation of alcohol intake, increased physical activity, reduced dietary intake of salt, saturated fats and cholesterol, and adequate dietary intake of potassium, calcium and magnesium. Each of these lifestyle changes has been shown to reduce blood pressure and improve overall well-being. However, there is no evidence for the routine prescription of calcium, potassium and magnesium supplements to aid the management of hypertension. Furthermore, excessive use of caffeine- and fructose-rich substances and beverages should be discouraged.

Every consultation with a hypertensive patient needs to be seen as an opportunity for health promotion and general cardiovascular risk assessment. Such assessment would include looking for hypertensive damage to target organs and involves a urine dipstick, a 12-lead ECG and blood tests for plasma glucose, electrolyte, creatinine and urea, and cholesterol levels. Education about and screening for cardiovascular disease at primary health centres should be increased.

Doctors working at primary healthcare level should regularly consider the use of specialist services if blood pressure remains elevated. Likewise, the publication of guidelines by specialists is not sufficient. There should be multiple opportunities for on-going education of all levels of doctors, on management of hypertension and for engagement between primary-level doctors and specialists. Medical outreach is one strategy for facilitating such interaction. Opportunities exist for engagement between healthcare professionals, academia, industry and civil society and need to be encouraged.^19^

## Conclusion

Detection and management of hypertension in South Africa remains sub-optimal. Doctors working alone have failed in their endeavour to improve the health of hypertensive patients and the prevention of premature mortality in this group of patients. The management of hypertension presents both complex challenges and opportunities. Doctors should not be content with their failure, but rather use their power and foresight, in collaboration with partners from various sectors, to deliver the promise of ‘a better health for all’.
